# Dynamics for a fractional-order predator-prey model with group defense

**DOI:** 10.1038/s41598-020-61468-3

**Published:** 2020-03-17

**Authors:** Bingnan Tang

**Affiliations:** Business school, Jiangsu University of Technology, Changzhou, 213001 P.R. China

**Keywords:** Animal disease models, Applied mathematics

## Abstract

In the present article, a new fractional order predator-prey model with group defense is put up. The dynamical properties such as the existence, uniqueness and boundness of solution, the stability of equilibrium point and the existence of Hopf bifurcation of the involved predator-prey model have been discussed. Firstly, we establish the sufficient conditions that guarantee the existence, uniqueness and boundness of solution by applying Lipschitz condition, inequality technique and fractional order differential equation theory. Secondly, we analyze the existence of various equilibrium points by basic mathematical analysis method and obtain some sufficient criteria which guarantee the locally asymptotically stability of various equilibrium points of the involved predator-prey model with the aid of linearization approach. Thirdly, the existence of Hopf bifurcation of the considered predator-prey model is investigated by using the Hopf bifurcation theory of fractional order differential equations. Finally, simulation results are presented to substantiate the theoretical findings.

## Introduction

It is well known that the interaction of predator species and prey species is an important topic in biology and mathematical ecology. Since the classical works of Lotka and Volterra^1,2^, the research on the dynamical behavior of predator-prey models has become a crucial research field for mathematicians and biologists. In the natural ecosystem, different biological populations take certain measures such as group defense, refuging, escaping and so on, to maintain their own survivals or search for food. To further grasp the law of interaction of predator species and prey species, many scholars pay much attention to the group defense mechanism of the prey. For example, Falconi *et al*.^3^ investigated the stability and global dynamic of a predator-prey model with group defense, Raw *et al*.^4^ reveal the complex dynamical behavior of prey-predator system with group defense, Xu *et al*.^5^ discussed the global dynamics of a predator-prey system with defense mechanism. In details, one can see^6–9^.

In 2013, Venturino and Petrovskii^10^ put up the following predator-prey model with group defense:

11 $$\left\{\begin{array}{l}{\dot{u}}_{1}(t)={\gamma }_{1}\left[1-\frac{{u}_{1}(t)}{\kappa }\right]{u}_{1}(t)-{\gamma }_{2}{u}_{1}^{\sigma }(t){u}_{2}(t),\\ {\dot{u}}_{2}(t)=-{\gamma }_{3}{u}_{2}(t)+{\gamma }_{2}{\gamma }_{4}{u}_{1}^{\sigma }(t){u}_{2}(t),\end{array}\right.$$

where *u*_1_ and *u*_2_ stand for the densities of prey and predator population, respectively. *γ*_1_ represents the logistic growth rate, *γ*_2_ stands for the search efficiency of predator for prey, *γ*_3_ stands for the mortality rate of predator species, *γ*_4_ stands for the biomass conversion coefficient, *κ* is the carrying capacity of the environment, and *σ* denotes aggregation efficiency. All the parameters *γ*_*i*_(*i* = 1, 2, 3, 4), *κ* are positive. Considering that the harvesting play an important role in describing the evolution process of a population, Kumar and Kharbanda^11^ introduced the Michaelis-Menten type harvesting into predator-prey model (1.1). Then they established the following predator-prey model with group defense and Michaelis-Menten type harvesting:

12 $$\left\{\begin{array}{l}{\dot{u}}_{1}(t)={\gamma }_{1}\left[1-\frac{{u}_{1}(t)}{\kappa }\right]{u}_{1}(t)-{\gamma }_{2}{u}_{1}^{\sigma }(t){u}_{2}(t)-\frac{{\rho }_{1}{\rho }_{2}{u}_{1}(t)}{{\rho }_{2}{\rho }_{3}+{\rho }_{4}{u}_{1}(t)},\\ {\dot{u}}_{2}(t)=-{\gamma }_{3}{u}_{2}(t)+{\gamma }_{2}{\gamma }_{4}{u}_{1}^{\sigma }(t){u}_{2}(t),\end{array}\right.$$

where *u*_1_ and *u*_2_ stand for the densities of prey and predator population, respectively, *ρ*_1_, *ρ*_2_ stand for the catchability parameter, the effort applied to harvest the prey species, respectively, *ρ*_3_ and *ρ*_4_ denote appropriate real constants, *γ*_*i*_(*i* = 1, 2, 3, 4), *κ* have the same implication as those in model (1.1). Based on the biological viewpoint, we assume that all the parameters are positive. Kumar and Kharbanda^11^ discussed detailedly the stability and Hopf bifurcation of model (1.2).

As is known to us, fractional calculus is a generalization of classical ordinary differentiation and integration^12–16^. For a long time, the fractional calculus has been maintained a slow development state due to the lack of practical background and technical means. Recently, fractional calculus has been found to be widely applied in numerous areas such as chemical engineering, viscoelasticity, biomedical Science, robotics, physics, mechanics and control science and so on^17–21^. Moreover, fractional-order differential equations can better describe the real objective phenomena than integer-order differential equations since they possess memory and hereditary natures of different materials and processes. Thus we think that the investigation on dynamical behavior of fractional-order differential equations have important theoretical significance and broad potential value.

## Model formulation

Considering that fractional calculus is a more suitable tool to describe memory and hereditary properties of numerous processes and materials and fractional-order equations are closely connected with memory for predator-prey systems^22,23^. Based on this viewpoint and inspired by the analysis in Section 1, we modify model (1.2) as the following fractional-order version:

21 $$\left\{\begin{array}{l}{D}^{\theta }{u}_{1}(t)={\gamma }_{1}\left[1-\frac{{u}_{1}(t)}{\kappa }\right]{u}_{1}(t)-{\gamma }_{2}{u}_{1}^{\sigma }(t){u}_{2}(t)-\frac{{\rho }_{1}{\rho }_{2}{u}_{1}(t)}{{\rho }_{2}{\rho }_{3}+{\rho }_{4}{u}_{1}(t)},\\ {D}^{\theta }{u}_{2}(t)=-{\gamma }_{3}{u}_{2}(t)+{\gamma }_{2}{\gamma }_{4}{u}_{1}^{\sigma }(t){u}_{2}(t),\end{array}\right.$$

where *D*^*θ*^ denotes the Caputo fractional derivative and 0 < *θ* < 1. We give the initial value of model (1.2) as follows: *u*_1_(*t*_0_) = *u*_10_ ≥ 0, *u*_2_(*t*_0_) = *u*_20_ ≥ 0. Denote ${R}_{+}^{2}=\{({u}_{1},{u}_{2})\in {R}^{2}| {u}_{1}\ \ge \ 0,{u}_{2}\ \ge \ 0\}$. $\Theta =\{({u}_{1},{u}_{2})\in {R}^{2}:\max \{| {u}_{1}| ,| {u}_{2}| \} < K\}$.

The key task of this manuscript is to handel two aspects: (1) seek the sufficient conditions to ensure the existence, uniqueness and boundness of solution, the stability of equilibrium point and the existence of Hopf bifurcation for system (2.1); (2) reveal the effect of fractional order on the stability and the existence of Hopf bifurcation of model (2.1).

The bright spots of the manuscript include the following four aspects:

- Based on the earlier works, a new fractional-order predator-prey model with group defense is established.
- Several sufficient criteria to ensure the existence, uniqueness and boundness of solution, the stability of equilibrium point and the existence of Hopf bifurcation of the involved predator-prey model are presented. The investigation reveals that fractional-order is an pivotal parameter in affecting the Hopf bifurcation of the involved fractional-order predator-prey model.
- Skillfully constructing a suitable Lyapunov function to prove the boundness of solution has achieved great success.
- The research approach of this paper will provide useful ideas for future investigation on numerous fractional-order differential systems.

This article is planned as follows. In Sect. 2, a fractional-order predator-prey model is established. In Sect. 3, few necessary definitions and lemmas are prepared. In Sect. 4, the existence, uniqueness and boundness of solution of model (2.1) is analyzed. In Sect. 5, the stability of equilibrium point of system (2.1) is discussed in detail. Hopf bifurcation analysis is carried out in Sect. 6. Some related computer simulations are given to check effectiveness of the main findings in Sect. 7. We ends the article with a simple conclusion in Sect. 8.

## Preliminary results

In this section, we give few definitions and lemmas.

**Definition 3.1**^24^ *The Caputo fractional derivative of order* *θ* *is defined as follows*:

$${D}^{\theta }h(\zeta )=\frac{1}{\Gamma (l-\theta )}{\int }_{{\zeta }_{0}}^{\zeta }\frac{{h}^{(l)}(\tau )}{{(\zeta -\tau )}^{\theta -l+1}}d\tau ,$$

*where* *h*(*ζ*) ∈ ([*ζ*_0_, *∞*), *R*), *ζ* ≥ *ζ*_0_ *and* *l* ∈ *N* *and satisfies* *l* − 1 ≤ *θ* < *l*.

Give the following Caputo fractional differential system:

3.1 $${D}^{\theta }U(t)=h(t,U(t)),U({t}_{0})={U}_{0},$$

where $U(t)={({u}_{1}(t),{u}_{2}(t),\cdots ,{u}_{n}(t))}^{T}\in {R}^{n}$ and $h:\left[{t}_{0},\infty \right)\times \Phi \to {R}^{n}$ is piecewise continuous function with respect to *t* and satisfies locally Lipschitz continuous in *U* on $\left[{t}_{0},\infty \right)\times \Phi ,\Phi \subset {R}^{n}.$

**Definition 3.2**^25,26^ *We say that* U^*^ *is an equilibrium point of system* (3.1) *if and only if* *h*(*t*, U^*^) = 0. 

**Lemma 3.1**^26,27^ *Let* *J*(U^*^) *be the Jacobian matrix of model* (3.1) *near* U^*^and *λ*_*i*_(*i* = 1, 2, ⋯ , *n*) *the eigenvalues of* *J*(U^*^). *If some eigenvalues of* *λ*_*i*_ *satisfy* $| arg({\lambda }_{i})|  > \frac{\theta \pi }{2}$ *and some other eigenvalues of* *λ*_*i*_ *satisfy* $| arg({\lambda }_{i})|  < \frac{\theta \pi }{2}$, *then the equilibrium point* U^*^ *is a saddle point*.

**Lemma 3.2**^26,27^ *Let* U^*^, *J*(U^*^) *be equilibrium point, Jacobian matrix of system* (3.1), *respectively, and* *λ*_*i*_ *be the eigenvalues of Jacobian matrix* *J*(U^*^) *of system* (2.1).

1. U^*^ *is locally asymptotically stable* ⇔ $| arg({\lambda }_{i})|  > \frac{\theta \pi }{2}$ *for all* *λ*_*i*_(*i* = 1, 2, ⋯ , *n*).
2. U^*^ *is stable* ⇔ $| arg({\lambda }_{i})| \ \ge \ \frac{\theta \pi }{2}$ *for all* *λ*_*i*_(*i* = 1, 2, ⋯ , *n*) *and the eigenvalues with* $| arg({\lambda }_{i})| =\frac{\theta \pi }{2}$ *have the same geometric multiplicity and algebraic multiplicity*.
3. U^*^ *is unstable* ⇔ ∃ *λ*_*i*_ *such that* $| arg({\lambda }_{i})|  < \frac{\theta \pi }{2}$.

**Lemma 3.3** ^26,28^*If the following conditions hold:*

1. *the Jacobian matrix* *J*(U^*^) *of system* (3.1) *near the equilibrium point has a pair of complex conjugate eigenvalues with positive real part*;
2. $$p(\theta )=\frac{\theta \pi }{2}-{\min }_{1\le i\le 2}| arg({\lambda }_{i})| =0;$$
3. $$\frac{dp(\theta )}{d\theta }{| }_{\theta ={\theta }_{0}}\ne 0,$$

*then system* (3.1) *undergoes a Hopf bifurcation near the equilibrium point* U^*^ *when* *θ* *crosses the critical value* *θ*_0_.

## Existence, uniqueness and boundness of solution

In this segment, we will discuss the existence, uniqueness and boundness of solution of model (2.1).

**Theorem 4.1** *System* (2.1) *with initial value* (*u*_10_, *u*_20_) *has a unique solution* (*u*_1_(*t*), *u*_2_(*t*)) ∈ Θ *for all* *t* ≥ *t*_0_. 

**Proof** Let

41 $$\left\{\begin{array}{l}{F}_{1}(u)={\gamma }_{1}\left[1-\frac{{u}_{1}(t)}{\kappa }\right]{u}_{1}(t)-{\gamma }_{2}{u}_{1}^{\sigma }(t){u}_{2}(t)-\frac{{\rho }_{1}{\rho }_{2}{u}_{1}(t)}{{\rho }_{2}{\rho }_{3}+{\rho }_{4}{u}_{1}(t)},{F}_{2}(u)=-{\gamma }_{3}{u}_{2}(t)+{\gamma }_{2}{\gamma }_{4}{u}_{1}^{\sigma }(t){u}_{2}(t),\end{array}\right.$$

where *u* = (*u*_1_, *u*_2_). Define a mapping as follows:

4.2 $$F(u)=({F}_{1}(u),{F}_{2}(u)).$$

$$\forall u,{\bar{u}}\in \Theta $$, one has

$$\begin{array}{lll}| | F(u)-F({\bar{u}})| |  & = & | {F}_{1}(u)-{F}_{1}({\bar{u}})| +| {F}_{2}(u)-{F}_{2}({\bar{u}})| \\  & = & \left|\left\{{\gamma }_{1}\left[1-\frac{{u}_{1}({t}_{1})}{\kappa }\right]{u}_{1}({t}_{1})-{\gamma }_{2}{u}_{1}^{\sigma }({t}_{1}){u}_{2}({t}_{1})-\frac{{\rho }_{1}{\rho }_{2}{u}_{1}({t}_{1})}{{\rho }_{2}{\rho }_{3}+{\rho }_{4}{u}_{1}({t}_{1})}\right.\right.\\  &  & \left.\left.-\,\left\{{\gamma }_{1}\left[1-\frac{{u}_{1}({t}_{2})}{\kappa }\right]{u}_{1}({t}_{2})-{\gamma }_{2}{u}_{1}^{\sigma }({t}_{2}){u}_{2}(t)-\frac{{\rho }_{1}{\rho }_{2}{u}_{1}({t}_{2})}{{\rho }_{2}{\rho }_{3}+{\rho }_{4}{u}_{1}({t}_{2})}\right\}\right\}\right|\\  &  & +| -{\gamma }_{3}{u}_{2}({t}_{1})+{\gamma }_{2}{\gamma }_{4}{u}_{1}^{\sigma }({t}_{1}){u}_{2}({t}_{1})-[-{\gamma }_{3}{u}_{2}({t}_{2})+{\gamma }_{2}{\gamma }_{4}{u}_{1}^{\sigma }({t}_{2}){u}_{2}({t}_{2})]| \\  &  & \le | {u}_{1}({t}_{1})-{u}_{1}({t}_{2})| \left|{\gamma }_{1}\left[1-\frac{{u}_{1}({t}_{1})}{\kappa }\right]-{\gamma }_{2}{u}_{1}^{\sigma -1}({t}_{1}){u}_{2}({t}_{1})-\frac{{\rho }_{1}{\rho }_{2}}{{\rho }_{2}{\rho }_{3}+{\rho }_{4}{u}_{1}({t}_{1})}\right|\\  &  & +\max \{| {u}_{1}({t}_{1})| ,| {u}_{1}({t}_{2})| \}\left|\left\{{\gamma }_{1}\left[1-\frac{{u}_{1}({t}_{1})}{\kappa }\right]-{\gamma }_{2}{u}_{1}{({t}_{1})}^{\sigma -1}{u}_{2}({t}_{1})-\frac{{\rho }_{1}{\rho }_{2}}{{\rho }_{2}{\rho }_{3}+{\rho }_{4}{u}_{1}({t}_{1})}\right\}\right.\\  &  & -\left.\left\{{\gamma }_{1}\left[1-\frac{{u}_{1}({t}_{2})}{\kappa }\right]-{\gamma }_{2}{u}_{1}^{\sigma -1}({t}_{2}){u}_{2}({t}_{2})-\frac{{\rho }_{1}{\rho }_{2}}{{\rho }_{2}{\rho }_{3}+{\rho }_{4}{u}_{1}({t}_{2})}\right\}\right|\\  &  & +| {u}_{2}({t}_{1})-{u}_{2}({t}_{2})| | -{\gamma }_{3}+{\gamma }_{2}{\gamma }_{4}{u}_{1}^{\sigma }({t}_{1})| \end{array}$$

43 $$\begin{array}{ll} & +\max \left\{| {u}_{1}({t}_{1})| ,| {u}_{1}({t}_{2})| \right\}| (-{\gamma }_{3}+{\gamma }_{2}{\gamma }_{4}{u}_{1}^{\sigma }({t}_{1}))-(-{\gamma }_{3}+{\gamma }_{2}{\gamma }_{4}{u}_{1}{({t}_{2})}^{\sigma })| \\  & \le | {u}_{1}({t}_{1})-{u}_{1}({t}_{2})| \left|{\gamma }_{1}\left[1-\frac{{u}_{1}({t}_{1})}{\kappa }\right]-{\gamma }_{2}{u}_{1}^{\sigma -1}({t}_{1}){u}_{2}({t}_{1})-\frac{{\rho }_{1}{\rho }_{2}}{{\rho }_{2}{\rho }_{3}+{\rho }_{4}{u}_{1}({t}_{1})}\right|\\  & +K\max \{| {u}_{1}({t}_{1})-{u}_{1}({t}_{2})| ,| {u}_{2}({t}_{1})-{u}_{2}({t}_{2})| \}\left(\frac{{\gamma }_{1}}{\kappa }+{\gamma }_{2}{K}^{\sigma -1}+\frac{{\rho }_{1}{\rho }_{4}}{{\rho }_{4}}\right)\\  & +| {u}_{2}({t}_{1})-{u}_{2}({t}_{2})| | -{\gamma }_{3}+{\gamma }_{2}{\gamma }_{4}{u}_{1}^{\sigma }({t}_{1})| \\  & +K\max \{| {u}_{1}({t}_{1})-{u}_{1}({t}_{2})| ,| {u}_{2}({t}_{1})-{u}_{2}({t}_{2})| \}2{\gamma }_{2}{\gamma }_{4}{K}^{\sigma }\\  & \le | {u}_{1}({t}_{1})-{u}_{1}({t}_{2})| \left|{\gamma }_{1}\left[1+\frac{K}{\kappa }\right]+{\gamma }_{2}{K}^{\sigma }-\frac{{\rho }_{1}{\rho }_{2}}{{\rho }_{2}{\rho }_{3}+{\rho }_{4}K}\right|\\  & +K\max \{| {u}_{1}({t}_{1})-{u}_{1}({t}_{2})| ,| {u}_{2}({t}_{1})-{u}_{2}({t}_{2})| \}\left(\frac{{\gamma }_{1}}{\kappa }+{\gamma }_{2}{K}^{\sigma -1}+\frac{{\rho }_{1}{\rho }_{4}}{{\rho }_{4}}\right)\\  & +| {u}_{2}({t}_{1})-{u}_{2}({t}_{2})| | -{\gamma }_{3}+{\gamma }_{2}{\gamma }_{4}{K}^{\sigma }| \\  & +K\max \{| {u}_{1}({t}_{1})-{u}_{1}({t}_{2})| ,| {u}_{2}({t}_{1})-{u}_{2}({t}_{2})| \}2{\gamma }_{2}{\gamma }_{4}{K}^{\sigma }\\  & \le \vartheta | u-{\bar{u}}| ,\end{array}$$

where

4.4 $$\vartheta =\max \left\{\left|{\gamma }_{1}\left(1+\frac{K}{\kappa }\right)+{\gamma }_{2}{K}^{\sigma }-\frac{{\rho }_{1}{\rho }_{2}}{{\rho }_{2}{\rho }_{3}+{\rho }_{4}K}\right|+K\left(\frac{{\gamma }_{1}}{\kappa }+{\gamma }_{2}{K}^{\sigma -1}+\frac{{\rho }_{1}{\rho }_{4}}{{\rho }_{4}}\right)+2{\gamma }_{2}{\gamma }_{4}{K}^{\sigma +1}\right\}.$$

Then *F*(*u*) satisfies the Lipschitz condition with respect to *u*. Thus we can conclude that system (2.1) with initial value (*u*_10_, *u*_20_) has a unique solution (*u*_1_(*t*), *u*_2_(*t*)) ∈ Θ for all *t* ≥ *t*_0_. The proof is completed.

**Theorem 4.2** *Every solution* (*u*_1_(*t*), *u*_2_(*t*)) *of system* (2.1) *with the initial condition* (*u*_1_(*t*_0_), *u*_2_(*t*_0_)) *is uniformly bounded and non-negative*.

**Proof** Let *U*(*t*) = *γ*_4_*u*_1_(*t*) + *u*_2_(*t*). Then

4.5 $$\begin{array}{lll}{D}^{\theta }U(t) & = & {\gamma }_{4}{D}^{\theta }{u}_{1}(t)+{D}^{\theta }(t){u}_{2}\\  & = & {\gamma }_{4}\left\{{\gamma }_{1}\left[1-\frac{{u}_{1}(t)}{\kappa }\right]{u}_{1}(t)-{\gamma }_{2}{u}_{1}^{\sigma }(t){u}_{2}(t)-\frac{{\rho }_{1}{\rho }_{2}{u}_{1}(t)}{{\rho }_{2}{\rho }_{3}+{\rho }_{4}{u}_{1}(t)}\right\}\\  &  & -\ {\gamma }_{3}{u}_{2}(t)+{\gamma }_{2}{\gamma }_{4}{u}_{1}^{\sigma }(t){u}_{2}(t)\\  & = & {\gamma }_{4}{\gamma }_{1}{u}_{1}(t)-\frac{{\gamma }_{4}{\gamma }_{1}}{\kappa }{u}_{1}^{2}(t)-\frac{{\gamma }_{4}{\rho }_{1}{\rho }_{2}{u}_{1}(t)}{{\rho }_{2}{\rho }_{3}+{\rho }_{4}{u}_{1}(t)}-{\gamma }_{3}{u}_{2}(t)\\  & = & {\gamma }_{4}{\gamma }_{1}{u}_{1}(t)-\frac{{\gamma }_{4}{\gamma }_{1}}{\kappa }{u}_{1}^{2}(t)-\frac{{\gamma }_{4}{\rho }_{1}{\rho }_{2}{u}_{1}(t)}{{\rho }_{2}{\rho }_{3}+{\rho }_{4}{u}_{1}(t)}-{\gamma }_{3}U(t)+{\gamma }_{3}{\gamma }_{4}{u}_{1}(t)\\  & \le  & ({\gamma }_{1}+{\gamma }_{3}){\gamma }_{4}{u}_{1}(t)-\frac{{\gamma }_{4}{\gamma }_{1}}{\kappa }{u}_{1}^{2}(t)-{\gamma }_{3}U(t)\end{array}$$

Then

4.6 $$\begin{array}{lll}{D}^{\theta }U(t)+{\gamma }_{3}U(t) & \le  & ({\gamma }_{1}+{\gamma }_{3}){\gamma }_{4}{u}_{1}(t)-\frac{{\gamma }_{4}{\gamma }_{1}}{\kappa }{u}_{1}^{2}(t)\\  & = & \frac{{\gamma }_{4}{\gamma }_{1}}{\kappa }{\left[{u}_{1}(t)-{\left(\frac{{\gamma }_{1}+{\gamma }_{3}}{2{\gamma }_{1}}\right)}^{2}\right]}^{2}+\frac{{\gamma }_{4}{({\gamma }_{1}+{\gamma }_{3})}^{2}}{4\kappa {\gamma }_{1}}\\  & \le  & \frac{{\gamma }_{4}{({\gamma }_{1}+{\gamma }_{3})}^{2}}{4\kappa {\gamma }_{1}}.\end{array}$$

In view of the results in^29^, one has

47 $$U(t)\le U(0){E}_{\theta }(-{\gamma }_{3}{t}^{\theta })+\frac{{\gamma }_{4}{({\gamma }_{1}+{\gamma }_{3})}^{2}}{4\kappa {\gamma }_{1}}{t}^{\theta }{E}_{\theta ,\theta +1}(-{\gamma }_{3}{t}^{\theta }),$$

where *E*_*θ*_ is the Mittag-Leffler function. In view of Lemma 5 and Corollary 6 of^29^, we have

4.8 $$U(t)\le \frac{{\gamma }_{4}{({\gamma }_{1}+{\gamma }_{3})}^{2}}{4\kappa {\gamma }_{1}},t\to \infty .$$

Thus every solution (*u*_1_(*t*), *u*_2_(*t*)) of system (2.1) with the initial condition (*u*_1_(*t*_0_), *u*_2_(*t*_0_)) is uniformly bounded.

## Equilibria and stability

In this section, we consider the equilibria and their stability. In order to obtain the equilibrium point of system (2.1), we can solve the following equations:

5.1 $$\left\{\begin{array}{l}{D}^{\theta }{u}_{1}(t)=0\\ {D}^{\theta }{u}_{2}(t)=0.\end{array}\right.$$

Namely,

5.2 $$\left\{\begin{array}{l}{\gamma }_{1}\left[1-\frac{{u}_{1}(t)}{\kappa }\right]{u}_{1}(t)-{\gamma }_{2}{u}_{1}^{\sigma }(t){u}_{2}(t)-\frac{{\rho }_{1}{\rho }_{2}{u}_{1}(t)}{{\rho }_{2}{\rho }_{3}+{\rho }_{4}{u}_{1}(t)}=0,\\ -\ {\gamma }_{3}{u}_{2}(t)+{\gamma }_{2}{\gamma }_{4}{u}_{1}^{\sigma }(t){u}_{2}(t)=0.\end{array}\right.$$

It is not difficult to obtain the following equilibria of system (2.1): ${E}_{1}(0,0),{E}_{2}({u}_{1}^{(1)},0),{E}_{3}({u}_{1}^{(2)},0),{E}_{4}({u}_{1\ast },{u}_{2\ast }),$ where

53 $$\left\{\begin{array}{lll}{u}_{1}^{(1)} & = & \frac{\left(1-\frac{{\rho }_{2}{\rho }_{3}}{{\rho }_{4}\kappa }\right)+\sqrt{{\left(1-\frac{{\rho }_{2}{\rho }_{3}}{{\rho }_{4}\kappa }\right)}^{2}-4\left(\frac{{\rho }_{1}{\rho }_{2}}{{\gamma }_{1}{\rho }_{4}\kappa }-\frac{{\rho }_{2}{\rho }_{3}}{{\rho }_{4}\kappa }\right)}}{2},\\ {u}_{1}^{(2)} & = & \frac{\left(1-\frac{{\rho }_{2}{\rho }_{3}}{{\rho }_{4}\kappa }\right)-\sqrt{{\left(1-\frac{{\rho }_{2}{\rho }_{3}}{{\rho }_{4}\kappa }\right)}^{2}-4\left(\frac{{\rho }_{1}{\rho }_{2}}{{\gamma }_{1}{\rho }_{4}\kappa }-\frac{{\rho }_{2}{\rho }_{3}}{{\rho }_{4}\kappa }\right)}}{2},\\ {u}_{1\ast } & = & {\left(\frac{{\gamma }_{3}}{{\gamma }_{2}{\gamma }_{3}{\kappa }^{\sigma }}\right)}^{\frac{1}{\sigma }},\\ {u}_{2\ast } & = & {u}_{1\ast }^{1-\sigma }\left(1-{u}_{1\ast }-\frac{{\rho }_{1}{\rho }_{2}}{{\gamma }_{1}({\rho }_{2}{\rho }_{3}+{u}_{1\ast }{\rho }_{4}\kappa )}\right).\end{array}\right.$$

**Theorem 5.1** *(1) System* (2.1) *always has the zero equilibrium point* *E*_1_(0, 0).

*(2) If* ${\left(1-\frac{{\rho }_{2}{\rho }_{3}}{{\rho }_{4}\kappa }\right)}^{2} < 4\left(\frac{{\rho }_{1}{\rho }_{2}}{{\gamma }_{1}{\rho }_{4}\kappa }-\frac{{\rho }_{2}{\rho }_{3}}{{\rho }_{4}\kappa }\right)$, *then system* (2.1) *has no boundary equilibrium points*.

*(3) If* ${\left(1-\frac{{\rho }_{2}{\rho }_{3}}{{\rho }_{4}\kappa }\right)}^{2}=4\left(\frac{{\rho }_{1}{\rho }_{2}}{{\gamma }_{1}{\rho }_{4}\kappa }-\frac{{\rho }_{2}{\rho }_{3}}{{\rho }_{4}\kappa }\right)$ *and* $\frac{{\rho }_{2}{\rho }_{3}}{{\rho }_{4}\kappa } < 1,$ *then system* (2.1) *has a unique boundary equilibrium points* ${ {\tilde{E}} }_{1}({ {\tilde{u}} }_{1}^{(1)},0)$, *where* ${ {\tilde{u}} }_{1}^{(1)}=\frac{1-\frac{{\rho }_{2}{\rho }_{3}}{{\rho }_{4}\kappa }}{2}$.

*(4) If* ${\left(1-\frac{{\rho }_{2}{\rho }_{3}}{{\rho }_{4}\kappa }\right)}^{2} > 4\left(\frac{{\rho }_{1}{\rho }_{2}}{{\gamma }_{1}{\rho }_{4}\kappa }-\frac{{\rho }_{2}{\rho }_{3}}{{\rho }_{4}\kappa }\right)$ *and* $\frac{{\rho }_{2}{\rho }_{3}}{{\rho }_{4}\kappa } < 1,$ *then system* (2.1) *has two boundary equilibrium points* ${ {\tilde{E}} }_{1}^{\ast }({ {\tilde{u}} }_{1}^{(1\ast )},0)$ *and* ${ {\tilde{E}} }_{1}^{\ast \ast }({ {\tilde{u}} }_{1}^{(1\ast \ast )},0)$ *where*

$${ {\tilde{u}} }_{1}^{(1\ast )}=\frac{\left(1-\frac{{\rho }_{2}{\rho }_{3}}{{\rho }_{4}\kappa }\right)+\sqrt{{\left(1-\frac{{\rho }_{2}{\rho }_{3}}{{\rho }_{4}\kappa }\right)}^{2}-4\left(\frac{{\rho }_{1}{\rho }_{2}}{{\gamma }_{1}{\rho }_{4}\kappa }-\frac{{\rho }_{2}{\rho }_{3}}{{\rho }_{4}\kappa }\right)}}{2}$$

*and*

$${ {\tilde{u}} }_{1}^{(1\ast \ast )}=\frac{\left(1-\frac{{\rho }_{2}{\rho }_{3}}{{\rho }_{4}\kappa }\right)-\sqrt{{\left(1-\frac{{\rho }_{2}{\rho }_{3}}{{\rho }_{4}\kappa }\right)}^{2}-4\left(\frac{{\rho }_{1}{\rho }_{2}}{{\gamma }_{1}{\rho }_{4}\kappa }-\frac{{\rho }_{2}{\rho }_{3}}{{\rho }_{4}\kappa }\right)}}{2}.$$

*(5) If* (1 − *u*_1*_)*γ*_1_(*ρ*_2_*ρ*_3_ + *u*_1*_ρ_4_*κ*) > *ρ*_1_*ρ*_2_, *then system* (2.1) *has the interior equilibrium pointE*_4_(*u*_1*_, *u*_2*_).

Since the proof of Theorem 5.1 is simple in view of (5.3). Here we omit it.

Next we discuss the stability of the equilibrium points. The Jacobian matrix of system (2.1) near the equilibrium point (*u*_1_, *u*_2_) is

5.4 $$J({u}_{1},{u}_{2})=\left[\begin{array}{ll}{\gamma }_{1}-\frac{2{\gamma }_{1}{u}_{1}}{\kappa }-{\gamma }_{2}\sigma {u}_{1}^{\sigma -1}{u}_{2}-\frac{{\rho }_{1}{\rho }_{2}^{2}{\rho }_{3}}{{({\rho }_{2}{\rho }_{3}+{\rho }_{4}{u}_{1})}^{2}} & -{\gamma }_{2}{u}_{1}^{\sigma }\\ \sigma {\gamma }_{2}{\gamma }_{4}{u}_{1}^{\sigma -1}{u}_{2} & -{\gamma }_{3}+{\gamma }_{2}{\gamma }_{4}{u}_{1}^{\sigma }\\ \end{array}\right].$$

**Theorem 5.2** *The equilibrium point* *E*_1_(0, 0) *of system* (2.1) *is locally asymptotically stable if* ${\gamma }_{1}-\frac{{\rho }_{1}}{{\rho }_{3}} < 0$ *and is a saddle point if* ${\gamma }_{1}-\frac{{\rho }_{1}}{{\rho }_{3}} > 0$.

**Proof** In view of (5.4), one can get

5.5 $$J(0,0)=\left[\begin{array}{ll}{\gamma }_{1}-\frac{{\rho }_{1}}{{\rho }_{3}} & 0\\ 0 & -{\gamma }_{3}\\ \end{array}\right]$$

It follows that the eigenvalues of *J*(0, 0) are ${\lambda }_{1}={\gamma }_{1}-\frac{{\rho }_{1}}{{\rho }_{3}},{\lambda }_{2}=-{\gamma }_{3}.$ When ${\gamma }_{1}-\frac{{\rho }_{1}}{{\rho }_{3}} < 0$, then *λ*_1_ < 0, *λ*_2_ < 0 and hence *arg*(*λ*_*i*_) = *π*(*i* = 1, 2) and $| arg({\lambda }_{i})|  > \frac{\theta \pi }{2}(i=1,2)$. Thus the equilibrium point *E*_0_(0, 0) of system (2.1) is locally asymptotically stable if ${\gamma }_{1}-\frac{{\rho }_{1}}{{\rho }_{3}} < 0$. When ${\gamma }_{1}-\frac{{\rho }_{1}}{{\rho }_{3}} > 0$, then *λ*_1_ > 0, *λ*_2_ < 0 and hence *arg*(*λ*_1_) = 0 and $| arg({\lambda }_{1})|  < \frac{\theta \pi }{2}$. Thus the equilibrium point *E*_0_(0, 0) of system (2.1) is a saddle point if ${\gamma }_{1}-\frac{{\rho }_{1}}{{\rho }_{3}} > 0.$ The proof of Theorem 5.2 is completed.

**Theorem 5.3** *(1) The equilibrium point* ${E}_{2}({u}_{1}^{(1)},0)$ *of system* (2.1) *is locally asymptotically stable if* ${\gamma }_{1}-\frac{2{\gamma }_{1}{u}_{1}^{(1)}}{\kappa }-\frac{{\rho }_{1}{\rho }_{2}^{2}{\rho }_{3}}{{({\rho }_{2}{\rho }_{3}+{\rho }_{4}{u}_{1}^{(1)})}^{2}} < 0$ *and* $-{\gamma }_{3}+{\gamma }_{2}{\gamma }_{4}{({u}_{1}^{(1)})}^{\sigma } > 0$

*(2) The equilibrium point*${E}_{2}({u}_{1}^{(1)},0)$*of system* (2.1) *is a saddle point if* $\left({\gamma }_{1}-\frac{2{\gamma }_{1}{u}_{1}^{(1)}}{\kappa }-\frac{{\rho }_{1}{\rho }_{2}^{2}{\rho }_{3}}{{({\rho }_{2}{\rho }_{3}+{\rho }_{4}{u}_{1}^{(1)})}^{2}}\right)$ $(-{\gamma }_{3}+{\gamma }_{2}{\gamma }_{4}{({u}_{1}^{(1)})}^{\sigma }) < 0$.

*(3) The equilibrium point*${E}_{2}({u}_{1}^{(1)},0)$*of system* (2.1) *is a saddle point if* ${\gamma }_{1}-\frac{2{\gamma }_{1}{u}_{1}^{(1)}}{\kappa }-\frac{{\rho }_{1}{\rho }_{2}^{2}{\rho }_{3}}{{({\rho }_{2}{\rho }_{3}+{\rho }_{4}{u}_{1}^{(1)})}^{2}} > 0$*and* $-{\gamma }_{3}+{\gamma }_{2}{\gamma }_{4}{({u}_{1}^{(1)})}^{\sigma } > 0$.

**Proof** In view of (5.4), one can get

56 $$J({u}_{1}^{(1)},0)=\left[\begin{array}{cc}{\gamma }_{1}-\frac{2{\gamma }_{1}{u}_{1}^{(1)}}{\kappa }-\frac{{\rho }_{1}{\rho }_{2}^{2}{\rho }_{3}}{{({\rho }_{2}{\rho }_{3}+{\rho }_{4}{u}_{1}^{(1)})}^{2}} & -{\gamma }_{2}{({u}_{1}^{(1)})}^{\sigma }\\ 0 & -{\gamma }_{3}+{\gamma }_{2}{\gamma }_{4}{({u}_{1}^{(1)})}^{\sigma }\\ \end{array}\right]$$

It follows that the eigenvalues of $J({u}_{1}^{(1)},0)$ are ${\lambda }_{1}={\gamma }_{1}-\frac{2{\gamma }_{1}{u}_{1}^{(1)}}{\kappa }-\frac{{\rho }_{1}{\rho }_{2}^{2}{\rho }_{3}}{{({\rho }_{2}{\rho }_{3}+{\rho }_{4}{u}_{1}^{(1)})}^{2}},{\lambda }_{2}=-{\gamma }_{3}+{\gamma }_{2}{\gamma }_{4}{({u}_{1}^{(1)})}^{\sigma }.$ Under the assumptions of (1), (2) and (3) of Theorem 5.3, we can easily conclude that the conclusion of Theorem 5.3 holds true. The proof of Theorem 5.3 is completed.

In a same way, we get the following result on the equilibrium point ${E}_{3}({u}_{1}^{(2)},0)$ of system (2.1).

**Theorem 5.4** *(1) The equilibrium point* ${E}_{3}({u}_{1}^{(2)},0)$ *of system* (2.1) *is locally asymptotically stable if* ${\gamma }_{1}-\frac{2{\gamma }_{1}{u}_{1}^{(2)}}{\kappa }-\frac{{\rho }_{1}{\rho }_{2}^{2}{\rho }_{3}}{{({\rho }_{2}{\rho }_{3}+{\rho }_{4}{u}_{1}^{(2)})}^{2}} < 0$ and $-{\gamma }_{3}+{\gamma }_{2}{\gamma }_{4}{({u}_{1}^{(2)})}^{\sigma } > 0$

*(2) The equilibrium point* $${E}_{3}({u}_{1}^{(2)},0)$$ *of system*(2.1)*is a saddle point if* $$\left({\gamma }_{1}-\frac{2{\gamma }_{1}{u}_{1}^{(2)}}{\kappa }-\frac{{\rho }_{1}{\rho }_{2}^{2}{\rho }_{3}}{{({\rho }_{2}{\rho }_{3}+{\rho }_{4}{u}_{1}^{(2)})}^{2}}\right)$$$$(-{\gamma }_{3}+{\gamma }_{2}{\gamma }_{4}{({u}_{1}^{(2)})}^{\sigma }) < 0$$.

*(3) The equilibrium point* ${E}_{3}({u}_{1}^{(2)},0)$ *of system* (2.1) *is a saddle point if* ${\gamma }_{1}-\frac{2{\gamma }_{1}{u}_{1}^{(2)}}{\kappa }-\frac{{\rho }_{1}{\rho }_{2}^{2}{\rho }_{3}}{{({\rho }_{2}{\rho }_{3}+{\rho }_{4}{u}_{1}^{(2)})}^{2}} > 0$*and* $-{\gamma }_{3}+{\gamma }_{2}{\gamma }_{4}{({u}_{1}^{(2)})}^{\sigma } > 0$.

**Theorem 5.5** *(1) If the one of following inequalities is satisfied: (a)* *α* ≤ 0 *(b)* *α* > 0, *α*^2^ − 4*β* < 0 *and* $\frac{\sqrt{4\beta -{\alpha }^{2}}}{\alpha } > \tan \frac{\theta \pi }{2}$. *Then the equilibrium point* *E*_4_(*u*_1*_, *u*_2*_) *of system* (2.1) *is locally asymptotically stable*.

**Proof** In view of (5.4), we have

57 $$J({u}_{1\ast },{u}_{2\ast })=\left[\begin{array}{ll}{\gamma }_{1}-\frac{2{\gamma }_{1}{u}_{1\ast }}{\kappa }-{\gamma }_{2}\sigma {u}_{1\ast }^{\sigma -1}{u}_{2\ast }-\frac{{\rho }_{1}{\rho }_{2}^{2}{\rho }_{3}}{{({\rho }_{2}{\rho }_{3}+{\rho }_{4}{u}_{1\ast })}^{2}} & -{\gamma }_{2}{u}_{1\ast }^{\sigma }\\ \sigma {\gamma }_{2}{\gamma }_{4}{u}_{1\ast }^{\sigma -1}{u}_{2\ast } & -{\gamma }_{3}+{\gamma }_{2}{\gamma }_{4}{u}_{1\ast }^{\sigma }\\ \end{array}\right].$$

Let

58 $$\left\{\begin{array}{l}{a}_{11}={\gamma }_{1}-\frac{2{\gamma }_{1}{u}_{1\ast }}{\kappa }-{\gamma }_{2}\sigma {u}_{1\ast }^{\sigma -1}{u}_{2\ast }-\frac{{\rho }_{1}{\rho }_{2}^{2}{\rho }_{3}}{{({\rho }_{2}{\rho }_{3}+{\rho }_{4}{u}_{1\ast })}^{2}},\\ {a}_{12}=-{\gamma }_{2}{u}_{1\ast }^{\sigma },\\ {a}_{21}=\sigma {\gamma }_{2}{\gamma }_{4}{u}_{1\ast }^{\sigma -1}{u}_{2\ast },\\ {a}_{22}=-{\gamma }_{3}+{\gamma }_{2}{\gamma }_{4}{u}_{1\ast }^{\sigma }.\end{array}\right.$$

Then (5.7) becomes

59 $$J({u}_{1\ast },{u}_{2\ast })=\left[\begin{array}{ll}{a}_{11} & {a}_{12}\\ {a}_{21} & {a}_{22}\end{array}\right].$$

The eigenvalues of *J*(*u*_1*_, *u*_2*_) are

5.10 $${\lambda }_{1}=\frac{\alpha +\sqrt{{\alpha }^{2}-4\beta }}{2},{\lambda }_{2}=\frac{\alpha -\sqrt{{\alpha }^{2}-4\beta }}{2},$$

where

5.11 $$\alpha ={a}_{11}+{a}_{22},\beta ={a}_{11}{a}_{22}-{a}_{12}{a}_{21}.$$

If *α* ≤ 0, then we consider three cases:

(i) If *α* = 0, then the eigenvalues of *J*(*u*_1*_, *u*_2*_) are a pair of complex conjugate *λ*_1_ and ${\bar{\lambda }}_{1}$. Hence $Re({\lambda }_{1})=Re({\bar{\lambda }}_{1})=0$ and $arg({\lambda }_{1})=\frac{\pi }{2},arg({\bar{\lambda }}_{1})=-\frac{\pi }{2}$. Thus $| arg({\lambda }_{1})|  > \frac{\theta \pi }{2}$ and $| arg({\bar{\lambda }}_{1})|  > \frac{\theta \pi }{2}$. In view of Lemma 3.2, we can conclude that the equilibrium point *E*_4_(*u*_1*_, *u*_2*_) of system (2.1) is locally asymptotically stable.
(ii) If *α* < 0, *α*^2^ − 4*β* ≥ 0, then the both eigenvalues of *J*(*u*_1*_, *u*_2*_) are *λ*_1_ < 0 and *λ*_2_ < 0. Hence $| arg({\lambda }_{i})| =\frac{\pi }{2} > \frac{\theta \pi }{2}(i=1,2)$. In view of Lemma 3.2, we can conclude that the equilibrium point *E*_4_(*u*_1*_, *u*_2*_) of system (2.1) is locally asymptotically stable.
(iii) If *α* < 0, *α*^2^ − 4*β* < 0, then the eigenvalues of *J*(*u*_1*_, *u*_2*_) are a pair of complex conjugate *λ*_1_ and ${\bar{\lambda }}_{1}$. Hence $\,{\rm{Re}}\,({\lambda }_{1})=\,{\rm{Re}}\,({\bar{\lambda }}_{1}) < 0$ and $arg({\lambda }_{i}) > \frac{\pi }{2}$. In view of Lemma 3.2, we can conclude that the equilibrium point *E*_4_(*u*_1*_, *u*_2*_) of system (2.1) is locally asymptotically stable.

If $\alpha  > 0,{\alpha }^{2}-4\beta  < 0,\frac{\sqrt{4\beta -{\alpha }^{2}}}{\alpha } > \tan \frac{\theta \pi }{2}$, then the both eigenvalues of *J*(*u*_1*_, *u*_2*_) are *λ*_1_ and ${\bar{\lambda }}_{1}$ satisfy $\,{\rm{Re}}\,({\lambda }_{1})=\,{\rm{Re}}\,({\bar{\lambda }}_{1}) > 0,\,{\rm{Im}}\,({\lambda }_{1})=-{\rm{Im}}\,({\bar{\lambda }}_{1})=\frac{\sqrt{4\beta -{\alpha }^{2}}}{2} > 0.$ Hence

$$\frac{\,{\rm{Im}}\,({\lambda }_{1})}{\,{\rm{Re}}\,({\lambda }_{1})} > \tan \frac{\theta \pi }{2},-\frac{{\rm{Im}}\,({\bar{\lambda }}_{1})}{\,{\rm{Re}}\,({\bar{\lambda }}_{1})} > \tan \frac{\theta \pi }{2}.$$

Then $| arg({\lambda }_{1})|  > \frac{\alpha \pi }{2},| arg({\bar{\lambda }}_{1})|  > \frac{\alpha \pi }{2}.$ In view of Lemma 3.2, we can conclude that the equilibrium point *E*_4_(*u*_1*_, *u*_2*_) of system (2.1) is locally asymptotically stable.

**Theorem 5.6** *(1) If the one of following inequalities is satisfied: (a)* *α* > 0, *α*^2^ − 4*β* ≥ 0 (*b*) *α*^2^ − 4*β* < 0, *α* > 0 *and* $\frac{\sqrt{4\beta -{\alpha }^{2}}}{\alpha } < \tan \frac{\theta \pi }{2}$. *Then the equilibrium point* *E*_4_(*u*_1*_, *u*_2*_) *of system* (2.1) *is unstable*.

**Proof** In view of (5.4), we have

$$J({u}_{1\ast },{u}_{2\ast })=\left[\begin{array}{ll}{\gamma }_{1}-\frac{2{\gamma }_{1}{u}_{1\ast }}{\kappa }-{\gamma }_{2}\sigma {u}_{1\ast }^{\sigma -1}{u}_{2\ast }-\frac{{\rho }_{1}{\rho }_{2}^{2}{\rho }_{3}}{{({\rho }_{2}{\rho }_{3}+{\rho }_{4}{u}_{1\ast })}^{2}} & -{\gamma }_{2}{u}_{1\ast }^{\sigma }\\ \sigma {\gamma }_{2}{\gamma }_{4}{u}_{1\ast }^{\sigma -1}{u}_{2\ast } & -{\gamma }_{3}+{\gamma }_{2}{\gamma }_{4}{u}_{1\ast }^{\sigma }\end{array}\right].$$

Let

$$\left\{\begin{array}{l}{a}_{11}={\gamma }_{1}-\frac{2{\gamma }_{1}{u}_{1\ast }}{\kappa }-{\gamma }_{2}\sigma {u}_{1\ast }^{\sigma -1}{u}_{2\ast }-\frac{{\rho }_{1}{\rho }_{2}^{2}{\rho }_{3}}{{({\rho }_{2}{\rho }_{3}+{\rho }_{4}{u}_{1\ast })}^{2}},\\ {a}_{12}=-{\gamma }_{2}{u}_{1\ast }^{\sigma },\\ {a}_{21}=\sigma {\gamma }_{2}{\gamma }_{4}{u}_{1\ast }^{\sigma -1}{u}_{2\ast },\\ {a}_{22}=-{\gamma }_{3}+{\gamma }_{2}{\gamma }_{4}{u}_{1\ast }^{\sigma }.\end{array}\right.$$

Then (5.7) becomes

$$J({u}_{1\ast },{u}_{2\ast })=\left[\begin{array}{ll}{a}_{11} & {a}_{12}\\ {a}_{21} & {a}_{22}\end{array}\right].$$

The eigenvalues of *J*(*u*_1*_, *u*_2*_) are

$${\lambda }_{1}=\frac{\alpha +\sqrt{{\alpha }^{2}-4\beta }}{2},{\lambda }_{2}=\frac{\alpha -\sqrt{{\alpha }^{2}-4\beta }}{2},$$

where

5.12 $$\alpha ={a}_{11}+{a}_{22},\beta ={a}_{11}{a}_{22}-{a}_{12}{a}_{21}.$$

(i) If *α* > 0, *α*^2^ − 4*β*≥0, then the both eigenvalues of *J*(*u*_1*_, *u*_2*_) are *λ*_1_ > 0 and *λ*_2_ > 0. Hence $| arg({\lambda }_{i})|  < \frac{\theta \pi }{2}(i=1,2)$. In view of Lemma 3.2, we can conclude that the equilibrium point *E*_4_(*u*_1*_, *u*_2*_) of system (2.1) is unstable.

(ii) If *α* > 0, *α*^2^ − 4*β* < 0 and $\frac{\sqrt{4\beta -{\alpha }^{2}}}{\alpha } < \tan \frac{\theta \pi }{2}$, then the eigenvalues of *J*(*u*_1*_, *u*_2*_) are a pair of complex conjugate *λ*_1_ and ${\bar{\lambda }}_{1}$. Hence $\,{\rm{Im}}\,({\lambda }_{1})=-{\rm{Im}}\,({\bar{\lambda }}_{1}) > 0$, $\,{\rm{Re}}\,({\lambda }_{1})=\,{\rm{Re}}\,({\bar{\lambda }}_{1})=\alpha  > 0.$ Then

$$\frac{\,{\rm{Im}}\,({\lambda }_{1})}{\,{\rm{Re}}\,({\lambda }_{1})} < \tan \frac{\theta \pi }{2},-\frac{\,{\rm{Im}}\,({\bar{\lambda }}_{1})}{\,{\rm{Re}}\,({\bar{\lambda }}_{1})} < \tan \frac{\theta \pi }{2}.$$

Thus $| arg({\lambda }_{1})|  < \frac{\alpha \pi }{2},| arg({\bar{\lambda }}_{1})|  < \frac{\alpha \pi }{2}.$ In view of Lemma 3.2, we can conclude that the equilibrium point *E*_4_(*u*_1*_, *u*_2*_) of system (2.1) is unstable.

## Bifurcation analysis

In this section, we will establish the sufficient condition that guarantees the existence of Hopf bifurcation of system (2.1).

**Theorem 6.1** *If* *α*^2^ − 4*β* > 0 *and* *α* > 0, *then a Hopf bifurcation of system* (2.1) *will appear around* *E*_4_(*u*_1*_, *u*_2*_) *when the fractional order* *θ* *crosses the critical value* ${\theta }_{0}=\frac{2}{\pi }\arctan \frac{\sqrt{| {\alpha }^{2}-4\beta | }}{\alpha }$.

**Proof** Denote ${\vartheta }=\frac{\alpha }{2}$ and $\psi =\frac{\sqrt{| {\alpha }^{2}-4\beta | }}{2}$. In view of the assumption *α* > 0, one get *ϑ* > 0. By the assumption *α*^2^ − 4*β* > 0 and (4.10), the the eigenvalues of *J*(*u*_1*_, *u*_2*_) of system (2.1) are a pair of complex conjugate *λ*_1,2_ = *ϑ* ± *iψ*. Next, $p({\theta }_{0})=\frac{{\theta }_{0}\pi }{2}-{\min }_{1\le i\le 2}| arg({\lambda }_{i})| =\frac{{\theta }_{0}\pi }{2}-arg\left(\frac{\psi }{\vartheta }\right)=arg\left(\frac{\psi }{\vartheta }\right)-arg\left(\frac{\psi }{\vartheta }\right)=0.$ Finally, $\frac{dp(\theta )}{d\theta }{| }_{\theta ={\theta }_{0}}=\frac{\pi }{2}\ne 0.$ In view of Lemma 3.3, we can conclude that a Hopf bifurcation of system (2.1) will appear around *E*_4_(*u*_1*_, *u*_2*_) when the fractional order *θ* crosses the critical value ${\theta }_{0}=\frac{2}{\pi }\arctan \frac{\sqrt{| {\alpha }^{2}-4\beta | }}{\alpha }$. The proof of Theorem 6.1 is completed.

**Remark 6.1** *In*^11^, *the authors studied the stability, Hopf bifurcation and chaotic behavior of integer order predator-pry system. In this article, we mainly focus on the existence, uniqueness and boundness of solution, the stability of equilibrium point and the existence of Hopf bifurcation of fractional order predator-prey model. The research method and theoretical findings are different from those in*^11^. *According to this viewpoint, the results of this paper complete the works of Kumar and Kharbanda*^11^.

## Numerical simulation

**Figure 1:**
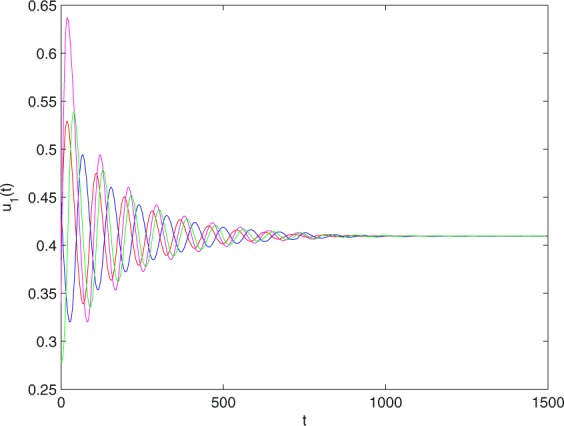
The trajectories of system (7.1) with *γ*_1_ = 1, *γ*_2_ = 1, *γ*_3_ = 0.2, *γ*_4_ = 0.25, *κ* = 1, *σ* = 0.25, *ρ*_1_ = 0.2, *ρ*_2_ = 1, *ρ*_3_ = 0.3, *ρ*_4_ = 1, *θ* = 0.78. The equilibrium point (0.4096, 0.1580) of system (7.1) is asymptotically stable. The relation of *t* and *u*_1_(*t*).

**Figure 2:**
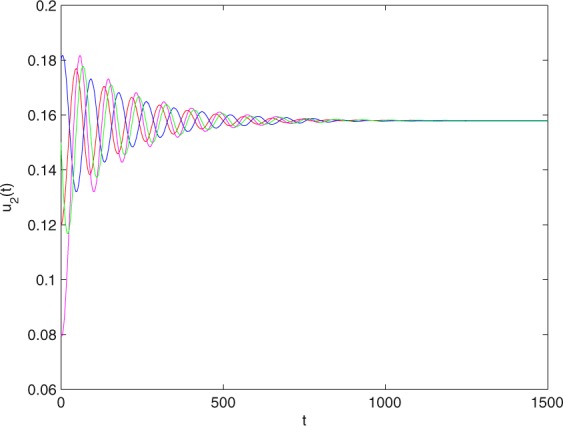
The trajectories of system (7.1) with *γ*_1_ = 1, *γ*_2_ = 1, *γ*_3_ = 0.2, *γ*_4_ = 0.25, *κ* = 1, *σ* = 0.25, *ρ*_1_ = 0.2, *ρ*_2_ = 1, *ρ*_3_ = 0.3, *ρ*_4_ = 1, *θ* = 0.78. The equilibrium point (0.4096, 0.1580) of system (7.1) is asymptotically stable. The relation of *t* and *u*_2_(*t*).

**Figure 3:**
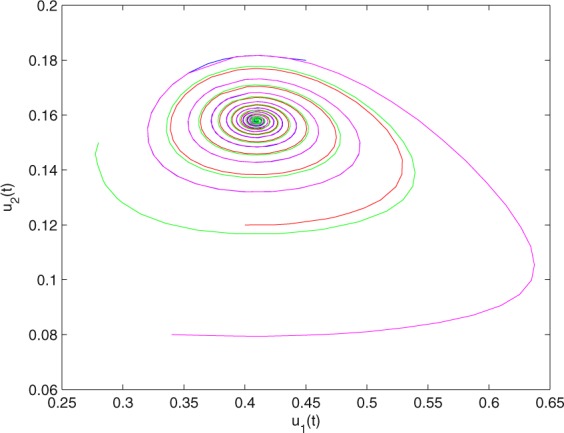
The trajectories of system (7.1) with *γ*_1_ = 1, *γ*_2_ = 1, *γ*_3_ = 0.2, *γ*_4_ = 0.25, *κ* = 1, *σ* = 0.25, *ρ*_1_ = 0.2, *ρ*_2_ = 1, *ρ*_3_ = 0.3, *ρ*_4_ = 1, *θ* = 0.78. The equilibrium point (0.4096, 0.1580) of system (7.1) is asymptotically stable. The relation of *u*_1_(*t*) and *u*_2_(*t*).

**Figure 4:**
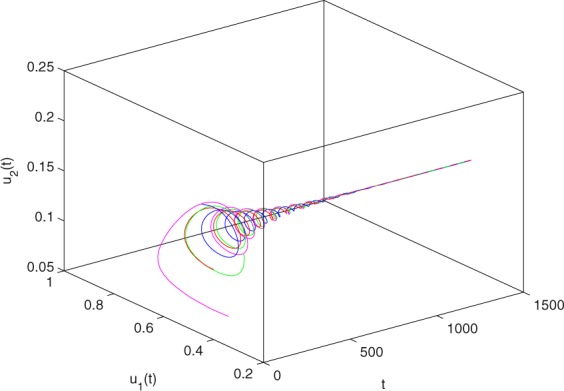
The trajectories of system (7.1) with *γ*_1_ = 1, *γ*_2_ = 1, *γ*_3_ = 0.2, *γ*_4_ = 0.25, *κ* = 1, *σ* = 0.25, *ρ*_1_ = 0.2, *ρ*_2_ = 1, *ρ*_3_ = 0.3, *ρ*_4_ = 1, *θ* = 0.78. The equilibrium point (0.4096, 0.1580) of system (7.1) is asymptotically stable. The relation of *t*, *u*_1_(*t*) and *u*_2_(*t*).

**Figure 5:**
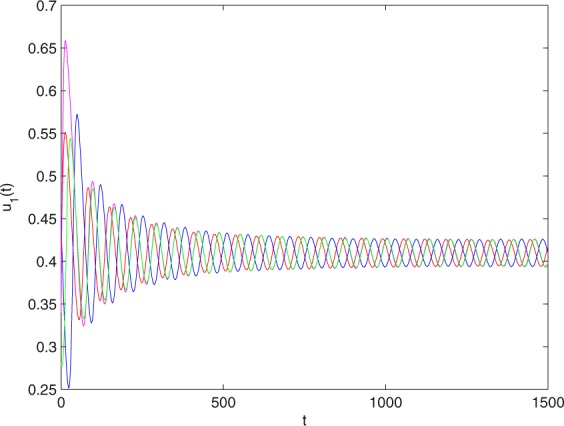
The trajectories of system (7.1) with *γ*_1_ = 1, *γ*_2_ = 1, *γ*_3_ = 0.2, *γ*_4_ = 0.25, *κ* = 1, *σ* = 0.25, *ρ*_1_ = 0.2, *ρ*_2_ = 1, *ρ*_3_ = 0.3, *ρ*_4_ = 1 and *θ* = 0.9 > *θ*_0_ = 0.83. Hopf bifurcation of system (7.1) appears from the equilibrium point (0.4096, 0.1580). The relation of *t* and *u*_1_(*t*).

**Figure 6:**
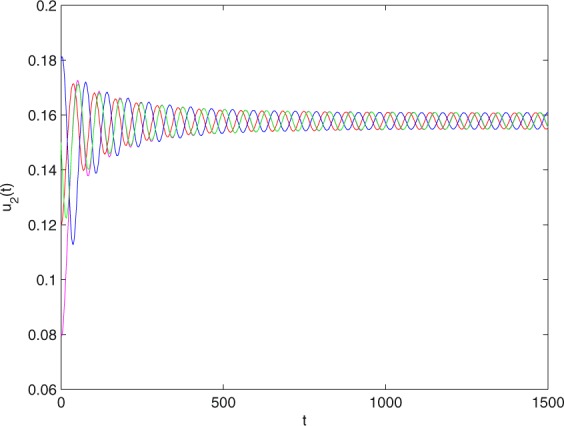
The trajectories of system (7.1) with *γ*_1_ = 1, *γ*_2_ = 1, *γ*_3_ = 0.2, *γ*_4_ = 0.25, *κ* = 1, *σ* = 0.25, *ρ*_1_ = 0.2, *ρ*_2_ = 1, *ρ*_3_ = 0.3, *ρ*_4_ = 1 and *θ* = 0.9 > *θ*_0_ = 0.83. Hopf bifurcation of system (7.1) appears from the equilibrium point (0.4096, 0.1580). The relation of *t* and *u*_2_(*t*).

**Figure 7:**
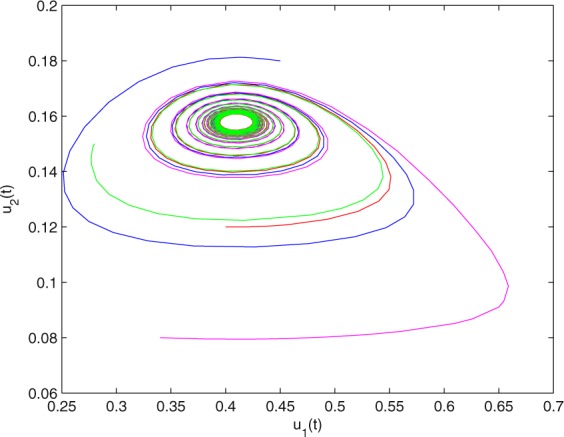
The trajectories of system (7.1) with *γ*_1_ = 1, *γ*_2_ = 1, *γ*_3_ = 0.2, *γ*_4_ = 0.25, *κ* = 1, *σ* = 0.25, *ρ*_1_ = 0.2, *ρ*_2_ = 1, *ρ*_3_ = 0.3, *ρ*_4_ = 1 and *θ* = 0.9 > *θ*_0_ = 0.83. Hopf bifurcation of system (7.1) appears from the equilibrium point (0.4096, 0.1580). The relation of *u*_1_(*t*) and *u*_2_(*t*).

**Figure 8:**
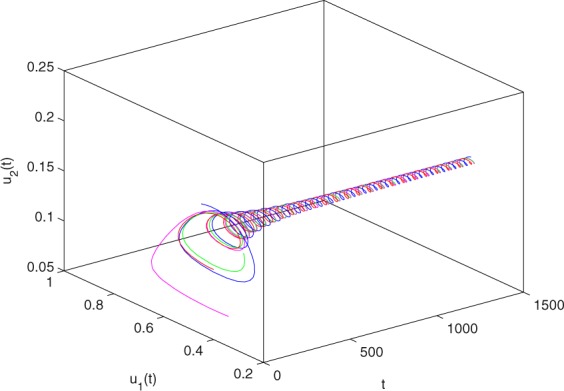
The trajectories of system (7.1) with *γ*_1_ = 1, *γ*_2_ = 1, *γ*_3_ = 0.2, *γ*_4_ = 0.25, *κ* = 1, *σ* = 0.25, *ρ*_1_ = 0.2, *ρ*_2_ = 1, *ρ*_3_ = 0.3, *ρ*_4_ = 1 and *θ* = 0.9 > *θ*_0_ = 0.83. Hopf bifurcation of system (7.1) appears from the equilibrium point (0.4096, 0.1580). The relation of *t*, *u*_1_(*t*) and *u*_2_(*t*).

**Figure 9:**
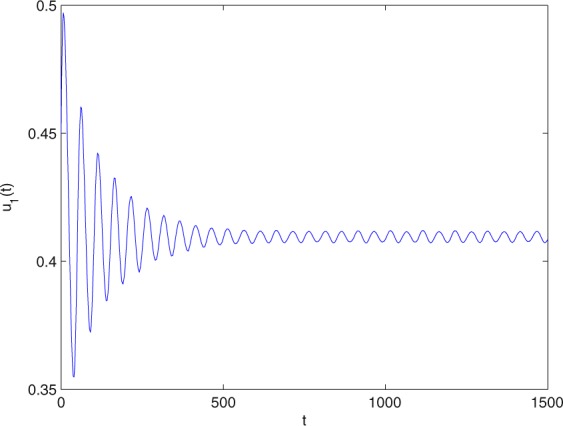
The trajectories of system (7.1) with ${\gamma }_{1}=0.999,{\gamma }_{2}=0.9,{\gamma }_{3}=0.2,{\gamma }_{4}=\frac{25}{9},\kappa =\frac{500}{499},\sigma =0.255,$ ${\rho }_{1}=0.2,{\rho }_{2}=1.1,{\rho }_{3}=0.25,{\rho }_{4}=1$and *θ* = 1. Hopf bifurcation appears near the equilibrium point (0.4168, 0.1465). The relation of *t* and *u*_1_(*t*).

**Figure 10:**
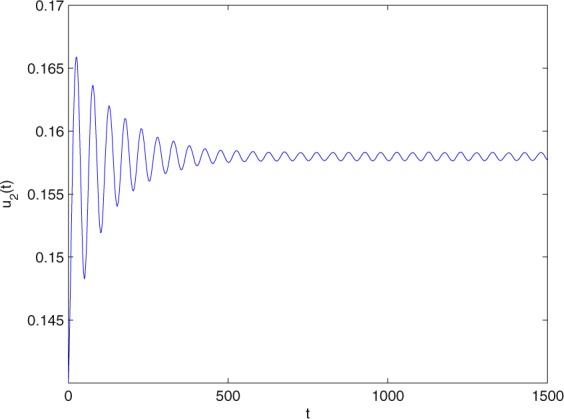
The trajectories of system (7.1) with ${\gamma }_{1}=0.999,{\gamma }_{2}=0.9,{\gamma }_{3}=0.2,{\gamma }_{4}=\frac{25}{9},\kappa =\frac{500}{499},\sigma =0.255,$ ${\rho }_{1}=0.2,{\rho }_{2}=1.1,{\rho }_{3}=0.25,{\rho }_{4}=1$and *θ* = 1. Hopf bifurcation appears near the equilibrium point (0.4168, 0.1465). The relation of *t* and *u*_2_(*t*).

**Figure 11:**
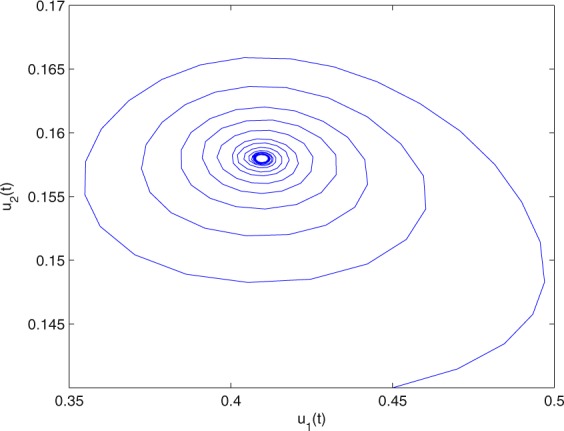
The trajectories of system (7.1) with ${\gamma }_{1}=0.999,{\gamma }_{2}=0.9,{\gamma }_{3}=0.2,{\gamma }_{4}=\frac{25}{9},\kappa =\frac{500}{499},\sigma =0.255,$ ${\rho }_{1}=0.2,{\rho }_{2}=1.1,{\rho }_{3}=0.25,{\rho }_{4}=1$and *θ* = 1. Hopf bifurcation appears near the equilibrium point (0.4168, 0.1465). The relation of *u*_1_(*t*) and *u*_2_(*t*).

**Figure 12:**
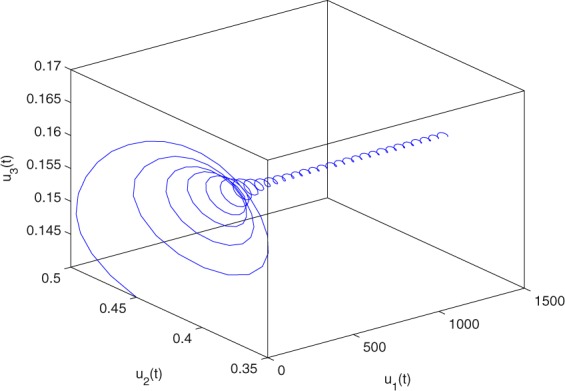
The trajectories of system (7.1) with ${\gamma }_{1}=0.999,{\gamma }_{2}=0.9,{\gamma }_{3}=0.2,{\gamma }_{4}=\frac{25}{9},\kappa =\frac{500}{499},\sigma =0.255,$ ${\rho }_{1}=0.2,{\rho }_{2}=1.1,{\rho }_{3}=0.25,{\rho }_{4}=1$and *θ* = 1. Hopf bifurcation appears near the equilibrium point (0.4168, 0.1465). The relation of *t*, *u*_1_(*t*) and *u*_2_(*t*).

**Example 7.1** We give the fractional-order system as follows:

71 $$\left\{\begin{array}{l}{D}^{\theta }{u}_{1}(t)=\left(1-{u}_{1}(t)\right){u}_{1}(t)-{u}_{1}^{0.25}(t){u}_{2}(t)-\frac{0.2{u}_{1}(t)}{0.3+{u}_{1}(t)},\\ {D}^{\theta }{u}_{2}(t)=-0.2{u}_{2}(t)+0.25{u}_{1}^{0.25}(t){u}_{2}(t),\end{array}\right.$$

where *γ*_1_ = 1, *γ*_2_ = 1, *γ*_3_ = 0.2, *γ*_4_ = 0.25, *κ* = 1, *σ* = 0.25, *ρ*_1_ = 0.2, *ρ*_2_ = 1, *ρ*_3_ = 0.3, *ρ*_4_ = 1. Obviously, system (7.1) has a unique coexistence equilibrium point *E*_3_(0.4096, 0.1580). By direct computation, one has *θ*_0_ = 0.83. Let *θ* = 0.78. We can easily check that all the assumptions of Theorem 5.4 and Theorem 6.1 are fulfilled. Thus the equilibrium point *E*_3_(0.4096, 0.1580) of system (7.1) is locally asymptotically stable. This fact is depicted in Figs. 1–4. When the parameter *θ* crosses the critical value *θ*_0_, then a Hopf bifurcation will appear. This result can be shown in Figs. 5–8 (here let *θ* = 0.9). If *θ* = 1 (integer order), a Hopf bifurcation appears near the equilibrium point *E*_3_(0.4096, 0.1580). This result can be illustrated in Figs. 9–12.

**Figure 13:**
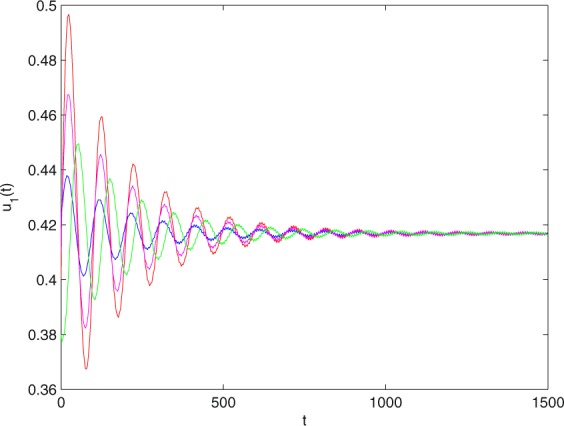
The trajectories of system (7.2) with ${\gamma }_{1}=0.999,{\gamma }_{2}=0.9,{\gamma }_{3}=0.2,{\gamma }_{4}=\frac{25}{9},\kappa =\frac{500}{499},\sigma =0.255,$ ${\rho }_{1}=0.2,{\rho }_{2}=1.1,{\rho }_{3}=0.25,{\rho }_{4}=1$and *θ* = 0.65. The equilibrium point (0.4168, 0.1465) of system (7.2) is asymptotically stable. The relation of *t* and *u*_1_(*t*).

**Figure 14:**
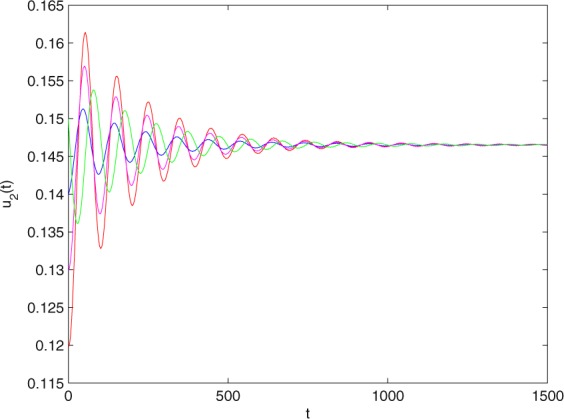
The trajectories of system (7.2) with ${\gamma }_{1}=0.999,{\gamma }_{2}=0.9,{\gamma }_{3}=0.2,{\gamma }_{4}=\frac{25}{9},\kappa =\frac{500}{499},\sigma =0.255,$ ${\rho }_{1}=0.2,{\rho }_{2}=1.1,{\rho }_{3}=0.25,{\rho }_{4}=1$and *θ* = 0.65. The equilibrium point (0.4168, 0.1465) of system (7.2) is asymptotically stable. The relation of *t* and *u*_2_(*t*).

**Figure 15:**
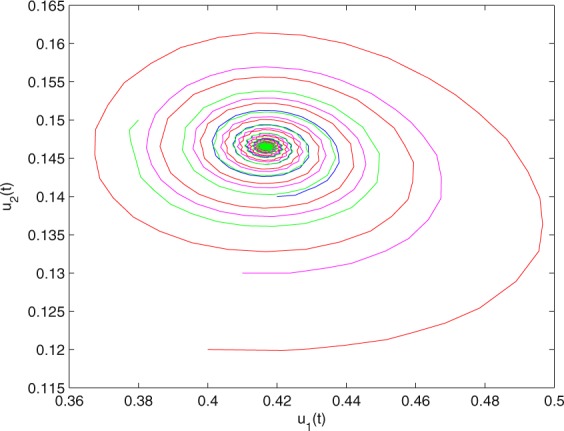
The trajectories of system (7.2) with ${\gamma }_{1}=0.999,{\gamma }_{2}=0.9,{\gamma }_{3}=0.2,{\gamma }_{4}=\frac{25}{9},\kappa =\frac{500}{499},\sigma =0.255,$ ${\rho }_{1}=0.2,{\rho }_{2}=1.1,{\rho }_{3}=0.25,{\rho }_{4}=1$and *θ* = 0.65. The equilibrium point (0.4168, 0.1465) of system (7.2) is asymptotically stable. The relation of *u*_1_(*t*) and *u*_2_(*t*).

**Figure 16:**
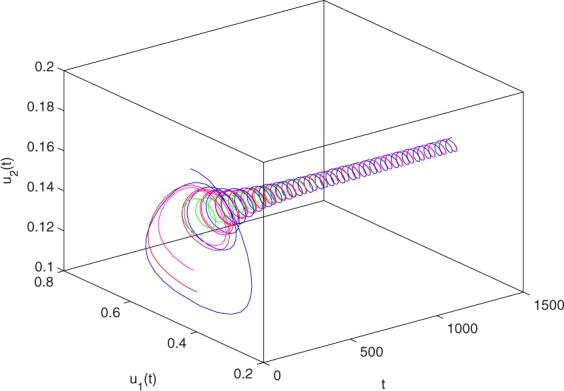
The trajectories of system (7.2) with ${\gamma }_{1}=0.999,{\gamma }_{2}=0.9,{\gamma }_{3}=0.2,{\gamma }_{4}=\frac{25}{9},\kappa =\frac{500}{499},\sigma =0.255,$ ${\rho }_{1}=0.2,{\rho }_{2}=1.1,{\rho }_{3}=0.25,{\rho }_{4}=1$and *θ* = 0.65. The equilibrium point (0.4168, 0.1465) of system (7.2) is asymptotically stable. The relation of *t*, *u*_1_(*t*) and *u*_2_(*t*).

**Figure 17:**
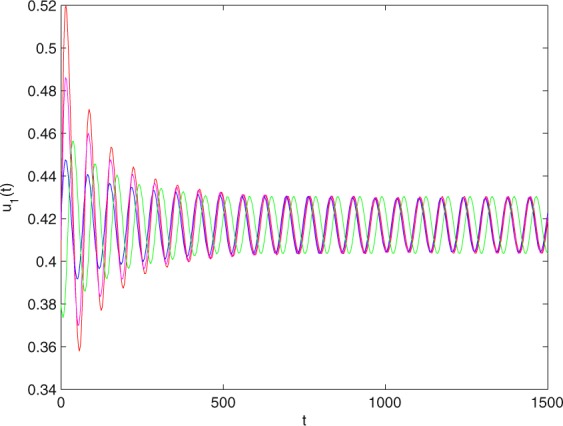
The trajectories of system (7.2) with ${\gamma }_{1}=0.999,{\gamma }_{2}=0.9,{\gamma }_{3}=0.2,{\gamma }_{4}=\frac{25}{9},\kappa =\frac{500}{499},\sigma =0.255,$ ${\rho }_{1}=0.2,{\rho }_{2}=1.1,{\rho }_{3}=0.25,{\rho }_{4}=1$ and *θ* = 0.823 > *θ*_0_ = 0.778. Hopf bifurcation of system (7.2) appears from the equilibrium point (0.4168, 0.1465). The relation of *t* and *u*_1_(*t*).

**Figure 18:**
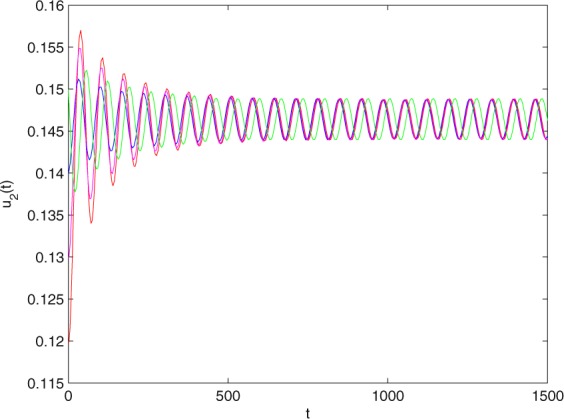
The trajectories of system (7.2) with ${\gamma }_{1}=0.999,{\gamma }_{2}=0.9,{\gamma }_{3}=0.2,{\gamma }_{4}=\frac{25}{9},\kappa =\frac{500}{499},\sigma =0.255,$ ${\rho }_{1}=0.2,{\rho }_{2}=1.1,{\rho }_{3}=0.25,{\rho }_{4}=1$and *θ* = 0.823 > *θ*_0_ = 0.778. Hopf bifurcation of system (7.2) appears from the equilibrium point (0.4168, 0.1465). The relation of *t* and *u*_2_(*t*).

**Figure 19:**
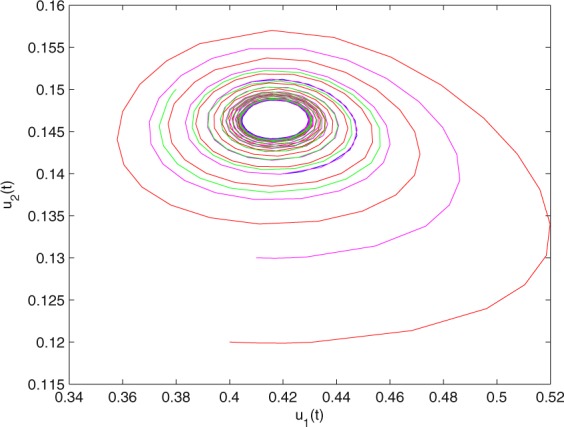
The trajectories of system (7.2) with ${\gamma }_{1}=0.999,{\gamma }_{2}=0.9,{\gamma }_{3}=0.2,{\gamma }_{4}=\frac{25}{9},\kappa =\frac{500}{499},\sigma =0.255,$ ${\rho }_{1}=0.2,{\rho }_{2}=1.1,{\rho }_{3}=0.25,{\rho }_{4}=1$ and *θ* = 0.823 > *θ*_0_ = 0.778. Hopf bifurcation of system (7.2) appears from the equilibrium point (0.4168, 0.1465). The relation of *u*_1_(*t*) and *u*_2_(*t*).

**Figure 20:**
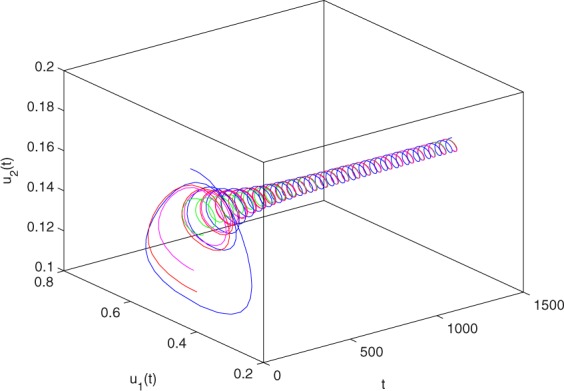
The trajectories of system (7.2) with ${\gamma }_{1}=0.999,{\gamma }_{2}=0.9,{\gamma }_{3}=0.2,{\gamma }_{4}=\frac{25}{9},\kappa =\frac{500}{499},\sigma =0.255,$ ${\rho }_{1}=0.2,{\rho }_{2}=1.1,{\rho }_{3}=0.25,{\rho }_{4}=1$ and *θ* = 0.823 > *θ*_0_ = 0.778. Hopf bifurcation of system (7.2) appears from the equilibrium point (0.4168, 0.1465). The relation of *t*, *u*_1_(*t*) and *u*_2_(*t*).

**Figure 21:**
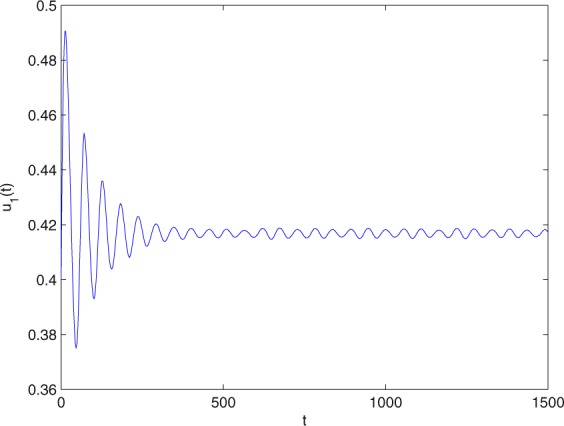
The trajectories of system (7.2) with ${\gamma }_{1}=0.999,{\gamma }_{2}=0.9,{\gamma }_{3}=0.2,{\gamma }_{4}=\frac{25}{9},\kappa =\frac{500}{499},\sigma =0.255,$ ${\rho }_{1}=0.2,{\rho }_{2}=1.1,{\rho }_{3}=0.25,{\rho }_{4}=1$ and *θ* = 1. Hopf bifurcation of system (7.2) appears neasr the equilibrium point (0.4168, 0.1465). The relation of *t* and *u*_1_(*t*).

**Figure 22:**
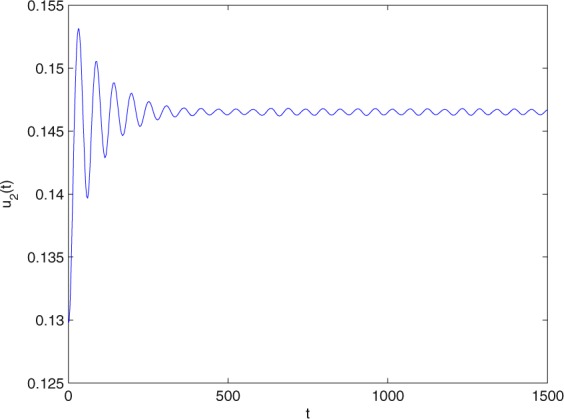
The trajectories of system (7.2) with ${\gamma }_{1}=0.999,{\gamma }_{2}=0.9,{\gamma }_{3}=0.2,{\gamma }_{4}=\frac{25}{9},\kappa =\frac{500}{499},\sigma =0.255,$ ${\rho }_{1}=0.2,{\rho }_{2}=1.1,{\rho }_{3}=0.25,{\rho }_{4}=1$ and *θ* = 1. Hopf bifurcation of system (7.2) appears neasr the equilibrium point (0.4168, 0.1465). The relation of *t* and *u*_2_(*t*).

**Figure 23:**
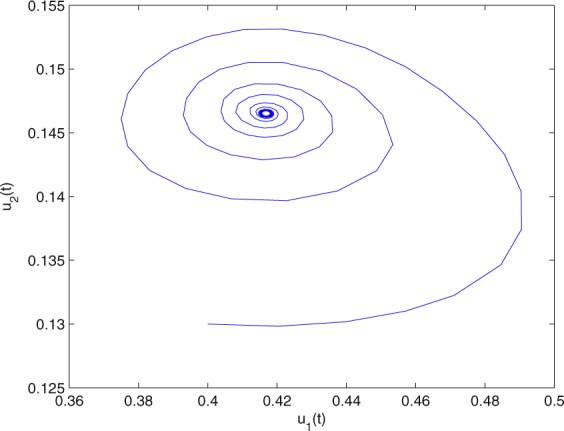
The trajectories of system (7.2) with ${\gamma }_{1}=0.999,{\gamma }_{2}=0.9,{\gamma }_{3}=0.2,{\gamma }_{4}=\frac{25}{9},\kappa =\frac{500}{499},\sigma =0.255,$ ${\rho }_{1}=0.2,{\rho }_{2}=1.1,{\rho }_{3}=0.25,{\rho }_{4}=1$ and *θ* = 1. Hopf bifurcation of system (7.2) appears neasr the equilibrium point (0.4168, 0.1465). The relation of *u*_1_(*t*) and *u*_2_(*t*).

**Figure 24:**
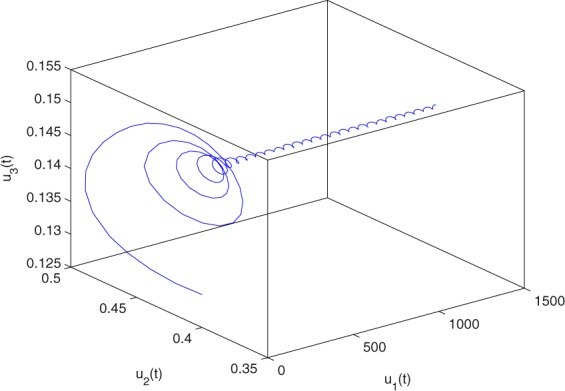
The trajectories of system (7.2) with ${\gamma }_{1}=0.999,{\gamma }_{2}=0.9,{\gamma }_{3}=0.2,{\gamma }_{4}=\frac{25}{9},\kappa =\frac{500}{499},\sigma =0.255,$ ${\rho }_{1}=0.2,{\rho }_{2}=1.1,{\rho }_{3}=0.25,{\rho }_{4}=1$ and *θ* = 1. Hopf bifurcation of system (7.2) appears neasr the equilibrium point (0.4168, 0.1465). The relation of *t*, *u*_1_(*t*) and *u*_2_(*t*).

**Example 7.2** We give the fractional-order system as follows:

72 $$\left\{\begin{array}{l}{D}^{\theta }{u}_{1}(t)=\left(0.999-0.998{u}_{1}(t)\right){u}_{1}(t)-{u}_{1}^{0.255}(t){u}_{2}(t)-\frac{0.22{u}_{1}(t)}{0.25+{u}_{1}(t)},\\ {D}^{\theta }{u}_{2}(t)=-0.2{u}_{2}(t)+0.25{u}_{1}^{0.255}(t){u}_{2}(t),\end{array}\right.$$

where ${\gamma }_{1}=0.999,{\gamma }_{2}=0.9,{\gamma }_{3}=0.2,{\gamma }_{4}=\frac{25}{9},\kappa =\frac{500}{499},\sigma =0.255,{\rho }_{1}=0.2,{\rho }_{2}=1.1,{\rho }_{3}=0.25,{\rho }_{4}=1.$ Obviously, system (7.2) has a unique coexistence equilibrium point *E*_3_(0.4168, 0.1465). By direct computation, one has *θ*_0_ = 0.778. Let *θ* = 0.65. We can easily check that all the assumptions of Theorem 5.5 and Theorem 6.1 are fulfilled. Thus the equilibrium point *E*_3_(0.4168, 0.1465) of system (7.2) is locally asymptotically stable. This fact is depicted in Figs. 13–16. When the parameter *θ* crosses the critical value *θ*_0_, then a Hopf bifurcation will appear. This result can be shown in Figs. 10–20 (here let *θ* = 0.823). If *θ* = 1 (integer order), a Hopf bifurcation appears near the equilibrium point *E*_3_(0.4168, 0.1465). This result can be illustrated in Figs. 21–24.

## Conclusions

In this article, we have discussed a fractional order predator-prey model with group defense. Some sufficient conditions that guarantee the the existence, uniqueness and boundness of solution, the stability of equilibrium point and the existence of Hopf bifurcation of the considered fractional order predator-prey model are established. The study shows that under some suitable parameter conditions, the various equilibrium points are locally asymptotically stable, when the parameter *θ* cross the critical value, the Hopf bifurcation will appear. The research also reveal that the fractional order has an important effect on the stability and the existence of Hopf bifurcation of the involved fractional order predator-prey model. At last, computer simulations are performed to illustrate the theoretical results. Numerical simulation results show that Hopf bifurcation value of Example 7.1 is *θ*_0_ = 0.83 and Hopf bifurcation value of Example 7.2 is *θ*_0_ = 0.778. The obtained results of this article can be applied to keep the coexistence of biological populations and maintain ecological balance. In addition, we must point out that time delay often exist in predator-prey system. But we still do not consider this case. We will deal with the dynamical behavior of fractional order delayed predator-prey models in the near future.
